# Supplementary material for: A comprehensive evaluation of cardiac amyloidosis epidemiology and diagnostics in French Guiana
Source: PLoS One. 2025 Jun 17;20(6):e0324955. doi: 10.1371/journal.pone.0324955 (PMC12173401; doi:10.1371/journal.pone.0324955)
Supplement: Attachment — Submitted filename: Responses to edidors&reviewers.Amylose.doc [file pone.0324955.s003.doc]

Responses to the editors and the reviewers

PONE-D-24-49901

"Unlocking Heart Health: Key Findings on Cardiac Amyloidosis in French Guiana Revealed!"

PLOS ONE

Dear Gbolahan Olatunji, M.D.
Academic Editor
PLOS ONE

**Please accept the revision of our original article, « Unlocking Heart Health: Key Findings on Cardiac Amyloidosis in French Guiana Revealed ». by Elenga, Narcisse; Desjardins B, Kénol F, Deschamps N, Alexis J, Mathien C, Boteko F, Yabeta G, Damy T, Inamo J. We are grateful of the reviewer's comments and have addressed them with the point-by-point response below. We truly believe that their constructive comments have further improved our article.**

Journal requirements:

When submitting your revision, we need you to address these additional requirements.

1. Please ensure that your manuscript meets PLOS ONE's style requirements, including those for file naming. The PLOS ONE style templates can be found at

https://journals.plos.org/plosone/s/file?id=wjVg/PLOSOne_formatting_sample_main_body.pdf and

[https://journals.plos.org/plosone/s/file?id=ba62/PLOSOne_formatting_sample_title_authors_affiliations.pdf](https://track.editorialmanager.com/CL0/https:%2F%2Fjournals.plos.org%2Fplosone%2Fs%2Ffile%3Fid=ba62%2FPLOSOne_formatting_sample_title_authors_affiliations.pdf/1/010f0194b8fc9bc5-159c6078-605c-4051-92f0-78b141b6d6ba-000000/gvLqDEqivxW1PvOEVF-prI4FufEyr5ICjaJV6OxEVFY=195)

**Thank you for this comment. We have revised our manuscript so that it meets PLOS ONE's style requirements.**

2. In the online submission form, you indicated that your data will be submitted to a repository upon acceptance.  We strongly recommend all authors deposit their data before acceptance, as the process can be lengthy and hold up publication timelines. Please note that, though access restrictions are acceptable now, your entire minimal dataset will need to be made freely accessible if your manuscript is accepted for publication. This policy applies to all data except where public deposition would breach compliance with the protocol approved by your research ethics board. If you are unable to adhere to our open data policy, please kindly revise your statement to explain your reasoning and we will seek the editor's input on an exemption.

**Thank you for this comment. We filed our database during this review process.**

3. We note that Figure 1 in your submission contain [map/satellite] images which may be copyrighted. All PLOS content is published under the Creative Commons Attribution License (CC BY 4.0), which means that the manuscript, images, and Supporting Information files will be freely available online, and any third party is permitted to access, download, copy, distribute, and use these materials in any way, even commercially, with proper attribution. For these reasons, we cannot publish previously copyrighted maps or satellite images created using proprietary data, such as Google software (Google Maps, Street View, and Earth). For more information, see our copyright guidelines: http://journals.plos.org/plosone/s/licenses-and-copyright.

We require you to either (1) present written permission from the copyright holder to publish these figures specifically under the CC BY 4.0 license, or (2) remove the figures from your submission:

1. You may seek permission from the original copyright holder of Figure 1  to publish the content specifically under the CC BY 4.0 license.

We recommend that you contact the original copyright holder with the Content Permission Form (http://journals.plos.org/plosone/s/file?id=7c09/content-permission-form.pdf) and the following text:

“I request permission for the open-access journal PLOS ONE to publish XXX under the Creative Commons Attribution License (CCAL) CC BY 4.0 (http://creativecommons.org/licenses/by/4.0/). Please be aware that this license allows unrestricted use and distribution, even commercially, by third parties. Please reply and provide explicit written permission to publish XXX under a CC BY license and complete the attached form.”

Please upload the completed Content Permission Form or other proof of granted permissions as an ""Other"" file with your submission.

In the figure caption of the copyrighted figure, please include the following text: “Reprinted from [ref] under a CC BY license, with permission from [name of publisher], original copyright [original copyright year].”

2. If you are unable to obtain permission from the original copyright holder to publish these figures under the CC BY 4.0 license or if the copyright holder’s requirements are incompatible with the CC BY 4.0 license, please either i) remove the figure or ii) supply a replacement figure that complies with the CC BY 4.0 license. Please check copyright information on all replacement figures and update the figure caption with source information. If applicable, please specify in the figure caption text when a figure is similar but not identical to the original image and is therefore for illustrative purposes only.

The following resources for replacing copyrighted map figures may be helpful:

USGS National Map Viewer (public domain): http://viewer.nationalmap.gov/viewer/

The Gateway to Astronaut Photography of Earth (public domain): http://eol.jsc.nasa.gov/sseop/clickmap/

Maps at the CIA (public domain): https://www.cia.gov/library/publications/the-world-factbook/index.html and https://www.cia.gov/library/publications/cia-maps-publications/index.html

NASA Earth Observatory (public domain): http://earthobservatory.nasa.gov/

Landsat: http://landsat.visibleearth.nasa.gov/

USGS EROS (Earth Resources Observatory and Science (EROS) Center) (public domain): http://eros.usgs.gov/#

Natural Earth (public domain): [http://www.naturalearthdata.com/](http://track.editorialmanager.com/CL0/http:%2F%2Fwww.naturalearthdata.com%2F/1/010f0194b8fc9bc5-159c6078-605c-4051-92f0-78b141b6d6ba-000000/Y6kT0bBs6Y0Tad0BcKpK5_d8vCj20C-YEExjEDlQPYE=195)

**We have removed Figure 1 from our manuscript as we are unable to obtain written permission from the copyright holder to publish it.**

Additional Editor Comments:

Please provide additional information on how you obtained a sample size of 47 patients. 
Which statistical methods did you use to make up for missing data, and how did you ensure standardization of data obtained from "private cardiologists"

**Thank you for this great comment. We have provided additional information on how we obtained a sample of 47 patients. We have also added details of the methods and provided the statistical methods used to adjust for missing data.**

Kindly improve the grammatical structure of the article

**We also had the whole manuscript proof-read by a recognised editor.**

NB: Please note that revision of your article does not guarantee acceptance

[Note: HTML markup is below. Please do not edit.]

Reviewers' comments:

Reviewer's Responses to Questions

**Comments to the Author**

1. Is the manuscript technically sound, and do the data support the conclusions?

The manuscript must describe a technically sound piece of scientific research with data that supports the conclusions. Experiments must have been conducted rigorously, with appropriate controls, replication, and sample sizes. The conclusions must be drawn appropriately based on the data presented.

Reviewer #1: Yes

Reviewer #2: No

Reviewer #3: Yes

Reviewer #4: Yes

2. Has the statistical analysis been performed appropriately and rigorously?

Reviewer #1: Yes

Reviewer #2: No

Reviewer #3: Yes

Reviewer #4: Yes

3. Have the authors made all data underlying the findings in their manuscript fully available?

The [PLOS Data policy](http://track.editorialmanager.com/CL0/http:%2F%2Fwww.plosone.org%2Fstatic%2Fpolicies.action%23sharing/1/010f0194b8fc9bc5-159c6078-605c-4051-92f0-78b141b6d6ba-000000/BO1mgsg_vuXsnk3XZ2gVn9a8dkIFpgrhPW9rc8ahLhY=195) requires authors to make all data underlying the findings described in their manuscript fully available without restriction, with rare exception (please refer to the Data Availability Statement in the manuscript PDF file). The data should be provided as part of the manuscript or its supporting information, or deposited to a public repository. For example, in addition to summary statistics, the data points behind means, medians and variance measures should be available. If there are restrictions on publicly sharing data—e.g. participant privacy or use of data from a third party—those must be specified.

Reviewer #1: Yes

Reviewer #2: Yes

Reviewer #3: Yes

Reviewer #4: Yes

4. Is the manuscript presented in an intelligible fashion and written in standard English?

PLOS ONE does not copyedit accepted manuscripts, so the language in submitted articles must be clear, correct, and unambiguous. Any typographical or grammatical errors should be corrected at revision, so please note any specific errors here.

Reviewer #1: Yes

Reviewer #2: No

Reviewer #3: Yes

Reviewer #4: Yes

5. Review Comments to the Author

Please use the space provided to explain your answers to the questions above. You may also include additional comments for the author, including concerns about dual publication, research ethics, or publication ethics. (Please upload your review as an attachment if it exceeds 20,000 characters)

Reviewer #1: "Unlocking Heart Health: Key Findings on Cardiac Amyloidosis in French Guiana Revealed!"

The authors performed a clinical multicenter retrospective study of Guianese patients with confirmed or suspected cardiac amyloidosis. 
The introduction is too long and should be more concise.

**We've rewritten the introduction and cut it in half.**
The introduction section should target the current study topic and related cardiac diseases. The following references might be helpful:
https://doi.org/10.1016/j.biopha.2017.04.033
<https://doi.org/10.1016/j.biopha.2023.114599>

**Thank you for these two references**
Did the author consider conducting an in vitro study using appropriate cardiomyocyte cell lines to confirm the current data?

**Great question. We are looking for a foreign team to collaborate on such a study.**
You should revise the manuscript for minor linguistic, grammatical, and style errors.

**Thank you. We had the whole manuscript proof-read by a recognised editor.**

You should revise the entire manuscript to ensure proper use of abbreviations.

**Thank done.**
Did the authors check their data for normality before applying the appropriate statistical test?

**Yes we did it**. **Thanks.**
The authors should explain and write the limitations of the current study.

**Yes we did it**. **Thanks**

Reviewer #2: The manuscript, titled "Unlocking Heart Health: Key Findings on Cardiac Amyloidosis in French Guiana," presents a retrospective, multicenter study evaluating the epidemiological characteristics and diagnostic pathways of cardiac amyloidosis in French Guiana. However, this study has major concerns and issues:

1. The introduction section is excessively lengthy and lacks a clear focus on the study's objectives. It includes superfluous details that detract from the main research question and reduce its overall clarity and impact.

**Thanks for your comments. We've rewritten the introduction and cut it in half,** **by focusing on the topic of the current study and related heart disease..**


2. The study reports a high rate of non-response or non-participation, which raises concerns about potential selection bias. This limitation significantly affects the robustness and generalizability of the findings, warranting further discussion and transparency in its potential implications.

**Thank you for this great comment. We have provided additional information on how we obtained our sample size. We have also added details of the methods and provided the statistical methods used to adjust for missing data. However, the limitations of the study were also discussed.**

Reviewer #3: I would like to thank the author for their contribution and manuscript. I have few comments:

**Thank you for comments**
1. Its better to replace "non-inclusion" to "exclusion" criteria.

**OK done**
2. Although cardiac amyloid is a rare entity and not commonly encountered. The sample size is very small especially over a period of 20 years of retrospective study.

**Thank you for this great comment. We have provided additional information on how we obtained our sample size. However, the limitations of the study were also discussed.** **However, 47 cases in 20 years in a current population of 300,000 seems to be a high prevalence for a rare disease.**

3. In the conclusion section its mention "with or suspected cardiac amyloid". Are there patients with suspected cardiac amyloid which has not been confirmed?

**Thank you for yur comment. Some changes have been made to the conclusion for clarity.**

Reviewer #4: Just 47 patients are included in the study, which is a small enough sample size to make generalizations. Furthermore, the majority of the study's patients (72.3%) are from Cayenne, which may introduce geographical bias.

**Thanks for your comments. The majority of patients in the study were from Cayenne, which may introduce a geographical bias. However, all patients with amyloidosis or suspected amyloidosis in French Guiana were referred to one of the hospital's cardiology departments.**

Justify the small sample size and talk about if it is representative of French Guiana's overall population.

**Thank you for this great comment. We have provided additional information on how we obtained our sample size. However, the limitations of the study were also discussed.** **However, 47 cases in 20 years in a current population of 300,000 seems to be a high prevalence for a rare disease.**

Provide methods for expanding patient recruitment, if possible.

**Now that we have established a regional amyloid team, a simplified and accessible diagnostic protocol, better training for healthcare professionals and a high level of public awareness of cardiac amyloidosis, the next step will be a prospective study to reduce bias and improve generalizability.**

Retrospective in nature, the study uses records from private and hospital cardiologists. Since only patients who have been diagnosed or are suspected of having amyloidosis within the healthcare system are included, this could introduce selection bias.

**Thanks for this great comment. We totally ahree with you. We have provided additional information on how we obtained our sample size. However, the limitations of the study were also discussed.**

Patients with cardiac amyloidosis are not compared to a control group of patients without the disease. It is challenging to determine whether specific risk factors are more prevalent in impacted individuals in the absence of this.

**We completely agree with you. We could do a case-control study as suggested. But first we wanted to do a cross-sectional study.**

It is challenging to evaluate the clinical relevance of reported differences because the study only offers p-values without providing effect sizes or confidence intervals (CIs).

**Confidence intervals have been added**

The study reports 38-month median survival, but does not compare this to other populations.

**We have compared survival with other populations**
The study's results are not sufficiently compared to earlier studies on cardiac amyloidosis in Latin America, the Caribbean, or French Guiana.

**We have compared survival with other studies, although there is little information on patients with cardiac amyloidosis in Latin American and Caribbean countries**.

Yours sincerely.
