# Supplementary material for: A comprehensive evaluation of cardiac amyloidosis epidemiology and diagnostics in French Guiana
Source: PLoS One. 2025 Jun 17;20(6):e0324955. doi: 10.1371/journal.pone.0324955 (PMC12173401; doi:10.1371/journal.pone.0324955)
Supplement: Attachment — Submitted filename: Responses to revewersR2.Amylose.doc [file pone.0324955.s004.doc]

Responses to the editors and the reviewers

PONE-D-24-49901

"Unlocking Heart Health: Key Findings on Cardiac Amyloidosis in French Guiana Revealed!"

PLOS ONE

Dear Gbolahan Olatunji, M.D.

Academic Editor

PLOS ONE

**Please accept the 2^nd^ revision of our original article, « Unlocking Heart Health: Key Findings on Cardiac Amyloidosis in French Guiana Revealed ». by Elenga, Narcisse; Desjardins B, Kénol F, Deschamps N, Alexis J, Mathien C, Boteko F, Yabeta G, Damy T, Inamo J. We are grateful of the reviewer's comments and have addressed them with the point-by-point response below. We truly believe that their constructive comments have further improved our article.**

Reviewer #2: The authors have not adequately addressed the comments, and significant concerns remain regarding selection bias, which may substantially impact the final conclusions

**Thank you for this comment. We discussed selection bias, compared our study with other studies in Latin American and Caribbean countries, and addressed limitations.**

Reviewer #3: I would like to thank the author for the revised manuscript. I would like to thank them for the changes made in relation to the provided comments and feedback. All comments were addressed with no shortcomings.

**Thank you so much**

Reviewer #4: The introduction contains excessive background information and lacks focus on the study objectives.

**Thank you for your comments. We've rewritten the introduction, focusing on the topic of the current study and related heart diseases, as well as the objectives of the study.**

The study includes only 47 patients over 20 years, raising concerns about selection bias, with 72.3% of patients from Cayenne, leading to geographical bias.

**Thank you for this comment. We have discussed these biases in the manuscript**.

Additionally, the absence of a comparison group of patients without cardiac amyloidosis makes it difficult to determine whether specific risk factors are truly more prevalent.

**This is a major limitation of our study, which we have highlighted. Thanks.**

The handling of missing data is unclear, and some results lack effect sizes and confidence intervals.

**Confidence intervals have been added but were not available for all variables.**

Furthermore, the Discussion section does not sufficiently compare findings with relevant studies from Latin America and the Caribbean, limiting the broader contextual relevance of the study.

**Thank you for this comment. We discussed selection bias, compared our study with other studies in Latin American and Caribbean countries, and addressed limitations.**
