# Supplementary material for: A comprehensive evaluation of cardiac amyloidosis epidemiology and diagnostics in French Guiana
Source: PLoS One. 2025 Jun 17;20(6):e0324955. doi: 10.1371/journal.pone.0324955 (PMC12173401; doi:10.1371/journal.pone.0324955)
Supplement: Attachment — Submitted filename: Responses to revewersR3.Amylose.doc [file pone.0324955.s005.doc]

Responses to the editors and the reviewers

PONE-D-24-49901

" A Comprehensive Evaluation of Cardiac Amyloidosis Epidemiology and Diagnostics in French Guiana"

PLOS ONE

Dear Gbolahan Olatunji, M.D.

Academic Editor

PLOS ONE

**Please accept the 3^rd^ revision of our original article, « A Comprehensive Evaluation of Cardiac Amyloidosis Epidemiology and Diagnostics in French Guiana». by Elenga, Narcisse; Desjardins B, Kénol F, Deschamps N, Alexis J, Mathien C, Boteko F, Yabeta G, Damy T, Inamo J. We are grateful of the reviewer's comments and have addressed them with the point-by-point response below. We truly believe that their constructive comments have further improved our article.**

**ACADEMIC EDITOR:**

Kindly include what type of article it is in the title and consider tweaking the title a little to be more scientific.

Thank you for this comment.

**The new title is : « A Comprehensive Evaluation of Cardiac Amyloidosis Epidemiology and Diagnostics in French Guiana »**

Journal Requirements:

Please review your reference list to ensure that it is complete and correct. If you have cited papers that have been retracted, please include the rationale for doing so in the manuscript text, or remove these references and replace them with relevant current references. Any changes to the reference list should be mentioned in the rebuttal letter that accompanies your revised manuscript. If you need to cite a retracted article, indicate the article’s retracted status in the References list and also include a citation and full reference for the retraction notice.

**We have reviewed and corrected all references**

Reviewer #5: I commend the authors’ work in revealing key findings on cardiac amyloidosis in French Guiana and their extensive revisions and responses to the previous reviewers’ comments.


I have a few comments:

- Some sentences/facts were not cited in the introduction section - please review and cite appropriately

**Thank you for this comment. We have added some sentences to describe Cardiad Amylioidosis**

- Authors should be consistent with the use of exclusion criteria vs. non-inclusion“ criteria—"exclusion criteria” is preferred, as initially noted by Reviewer 3. See pg 6/47 of “Amylose.GuyaneRevisedCLEAN(2)” and adjust

**OK corrected , thanks**

Reviewer #6:  Thank you for your paper.
The data was analyzed intelligently and clearly for interpretation.
I suggest that a heading of "LIMITATIONS" be included before the paragraph that outlines the study's limitations.

**We have added a title « Limitations » as suggested.**
